# Plasma arginase-1 as a predictive marker for early transarterial chemoembolization refractoriness in unresectable hepatocellular carcinoma

**DOI:** 10.3389/fonc.2022.1014653

**Published:** 2022-09-20

**Authors:** Wei-Li Xia, Shi-Jun Xu, Yuan Guo, Xiao-Hui Zhao, Hong-Tao Hu, Yan Zhao, Quan-Jun Yao, Lin Zheng, Dong-Yang Zhang, Chen-Yang Guo, Wei-Jun Fan, Hai-Liang Li

**Affiliations:** ^1^ Department of Minimal-Invasive Intervention, the Affiliated Cancer Hospital of Zhengzhou University & Henan Cancer Hospital, Zhengzhou, China; ^2^ Department of Minimally Invasive Interventional Radiology, Sun Yat-sen University Cancer Center, State Key Laboratory of Oncology in South China, Collaborative Innovation Cancer for Cancer Medicine, Guangzhou, China

**Keywords:** hepatocellular carcinoma, arg1, TACE refractoriness, prognostic factor, predictive models

## Abstract

**Objective:**

To explore the relationship between plasma arginase-1 (ARG1) and early transarterial chemoembolization (TACE) refractoriness in patients with hepatocellular carcinoma (HCC) and develop nomograms for predicting early TACE refractoriness.

**Methods:**

A total of 200 patients with HCC, treated with TACE, were included in the study, including 120 in the training set and 80 in the validation set. Pre-treatment enzyme-linked immunosorbent assay was used to detected the plasma ARG1 levels of the patient, and independent predictors of early TACE refractoriness were determined using a multivariate logistic regression model, based on which a predictive model was developed using a nomogram.

**Results:**

Risk of early TACE refractoriness was negatively correlated with plasma ARG1 levels, and multivariate logistic analysis showed tumour size (OR = 1.138, 95% CI = 1.006-1.288, P = 0.041), multiple tumors (OR=4.374, 95% CI = 1.189-16.089, P = 0.026), platelet count (OR = 0.990, 95% CI = 0.980-0.999, P = 0.036), and plasma ARG1 levels (OR = 0.209, 95% CI = 0.079-0.551, P = 0.002) to be independent prognostic factors for early TACE refractoriness.The AUC value for the nomogram of the training cohort was 0.786 (95% CI = 0.702–0.870), and the validation set AUC value was 0.833 (95% CI = 0.791-0.875).The decision curve analysis suggested that the nomogram had good clinical utility.

**Conclusion:**

High plasma ARG1 expression was associated with a lower incidence of early TACE refractoriness. The nomogram constructed based on four independent prognostic factors could facilitate an individualised prediction of the incidence of early TACE refractoriness.

## Introduction

Hepatocellular carcinoma (HCC) is estimated to be one of the most common malignant tumors in the world and one of the most common causes of cancer-related death (1, 2). Intrahepatic metastasis and multi-center origin are the biological characteristics of HCC, which has an insidious onset and a high degree of malignancy (3). According to the Barcelona Clinic Liver Cancer (BCLC) and European Society for Medical Oncology guidelines, transarterial chemoembolization (TACE) is the most important treatment option for patients with unresectable HCC in the early and middle stages (4–6); however, many years of clinical practice has shown proven that repeated TACE can weaken the therapeutic effect and cause TACE refractoriness due to its heterogeneity and the limitations of TACE surgery (7). Studies have found that in cases experiencing TACE refractoriness, the early use of combination therapy confers significant survival benefits and protection of liver function (8). The time required for identifying TACE refractoriness and the adjustment of the corresponding treatment strategy can have various effects on overall survival (OS) in patients with unresectable HCC. Accordingly, early detection of TACE refractoriness and transfer to systemic therapy can have important clinical effects (9).

Arginase-1 (ARG1), an enzyme that converts arginine to urea in the urea cycle, is mainly found in hepatocytes around the hilar of the liver. It can hydrolyze L-arginine, generate urea and L-ornithine, and detoxify ammonia. ARG1 is a more sensitive and specific marker of hepatocyte differentiation compared with other hepatocyte markers. Previous studies have shown that the expression level of ARG1 in liver tissues and paracancerous tissues is significantly higher than that in HCC tissues, and the disease-free survival (DFS) and overall survival (OS) of patients with HCC are related to the expression of ARG1, indicating that ARG1 may play a regulatory role in the occurrence and development of HCC (10, 11).

Previous studies had primarily focused on the relationship between TACE refractoriness and common imaging and laboratory indicators. In this study, by investigating the association between pre-treatment plasma ARG1 levels and early TACE refractoriness in patients with HCC, we aimed to identify patients who may show TACE refractoriness by developing a novel plasma ARG1-based nomogram.

## Materials and methods

### Patients

The study cohort comprised patients receiving TACE treatment in the Affiliated Cancer Hospital of Zhengzhou University from September 2017 to December 2020. The study was conducted in line with the guidelines of the Declaration of Helsinki and was approved by the Ethics Review Committee of the Affiliated Cancer Hospital of Zhengzhou University & Henan Cancer Hospital (approval number: 2016ct004). Due to the retrospective nature of the study, the ethics committee waived the requirement for informed consent. The data was analyzed anonymously. HCC was defined by pathological evidence or diagnosed using the non-invasive criteria of the European Association for the Study of the Liver and the American Association for the Study of Liver Diseases.

The training and validation cohorts were subjected to the same inclusion and exclusion criteria. The inclusion criteria were as follows: (1) Patients should be over 18 years old with at least one measurable target lesion on the liver; (2) Patients should show indications and no contraindication for TACE; (3) TACE performed as monotherapy; (4) Before the first TACE treatment, the status score of Eastern Cooperative Oncology Group performance should be 0; (5) Patients at BCLC stage A or B, on whom radical surgical resection or ablation could not be performed; (6) Patients with good liver function with Child-Pugh A or B grade; and (7) Patients with complete clinical data. The exclusion criteria were as follows: (1) Patients who have received systemic therapy such as radiotherapy, chemotherapy, targeted therapy, and immunotherapy, (2) Patients who have received surgery or radiofrequency ablation within 6 months after the first TACE; (3) Those with severe heart disease, or those with irrecoverable blood clotting disorder or renal dysfunction.

### TACE procedure

As reported previously (12), all TACE procedures were performed by at least two experienced interventional radiologists, and TACE was performed through the traditional femoral artery approach under local anesthesia. 5F RH catheters (Terumo, Tokyo, Japan) were first used for routine angiography, and then microcatheters (Terumo, Tokyo, Japan) were used for super selective arterial catheterization to enter the blood supply branch of the tumor. A mixed solution containing lipiodol (Laboratories Guerbet, Paris, France) and doxorubicin (Haizheng Pharmaceutical, Taizhou, China) was administered into the tumor-feeding vessels, followed by the injection of gelatin sponge particles (500 mm-700 mm; ALICON Dr. SCI&TEC Co., Ltd., Hangzhou, China) to supplement embolization until blood flow nearly ceased. The dose of doxorubicin was 50-70 mg and that of lipiodol was 5-20 ml; the specific dose should be adjusted according to the patient’s tumor number and size, liver function, blood vessel distribution, and body surface area.

### Definition of TACE refractoriness

As previously reported by the Japan Society of Hepatology (JSH), TACE refractoriness was mainly defined in terms of an insufficient response of the treated tumor or a progressive tumor response, indicated through the following observations: 1) viable lesions more than 50% after two or more TACE; 2) two or more intrahepatic lesions after TACE; 3) an increase in the extent of intrahepatic vascular invasion; 4) occurrence of extrahepatic metastasis; and 5) a continuous increase in the levels of tumor markers, following a temporary decrease in the levels after TACE (13). As had been reported previously, if the patient developed TACE refractoriness within 12 months after the initial TACE, we defined it as early TACE refractoriness (14).

If viable tumors were detected and liver function was adequate, TACE was repeated every 6–8 weeks. Follow-up included check-up for general health condition, liver function tests, routine blood investigations, and contrast-enhanced CT or MRI examination of the liver

### ARG1 measurements

Blood samples of the patients were collected before the first TACE; the plasma was then separated by centrifugation at 1000 × g for 10min at 4°C and stored at -80°C until ARG1 analyses. Human plasma ARG1 levels were quantified using an ELISA kit. Measurements were performed according to the manufacturer’s (Enzyme-linked Biotechnology Co., Ltd., Shanghai, China) instructions; the lowest and highest detectable levels of the kit were 0.9375 ng/mL and 30 ng/mL, respectively, and all samples were diluted 3-fold with the sample diluent prior to the experiment. Furthermore, 50 mL of both sample and standard were added to the corresponding wells, followed by the addition of 100 mL of horseradish peroxidase-labeled detection antibody to each well, and incubation at 37°C for 60 min. Thereafter, the liquid was discarded, and each well was washed five times with 350 µL of washing solution; 50 µL each of substrate A and B was added next and the mixture was incubated at 37°C for 15 min in the dark. Next, 50 mL of stop solution was added to each well, and using a plate reader (Thermos Fisher Scientific, China), absorbance was measured at 450 nm, after 15 min.

### Statistical analyses

SPSS (version 13.0; SPSS Inc., Chicago, IL, USA) was used for statistical analysis, and a binary logistic regression model was used to incorporate statistically significant variables into the multivariate analysis to identify predictors associated with the development of early TACE refractoriness. Nomograms were developed using R programming (Fundamentals of Statistical Computing version 4.1.2, Vienna, Austria), and the performance of each was evaluated using the consistency index (c-index). The c-index value ranges from 0.5 to 1, where a higher c-index indicates better predictive ability of the model. The value of the c-index ranged from 0.5 to 1. Decision curve analysis (DCA) was used to evaluate the accuracy of the model and net clinical benefits. Statistical significance was set at P < 0.05.

## Results

### ARG1 expression and cutoff value

**Figure 1:**
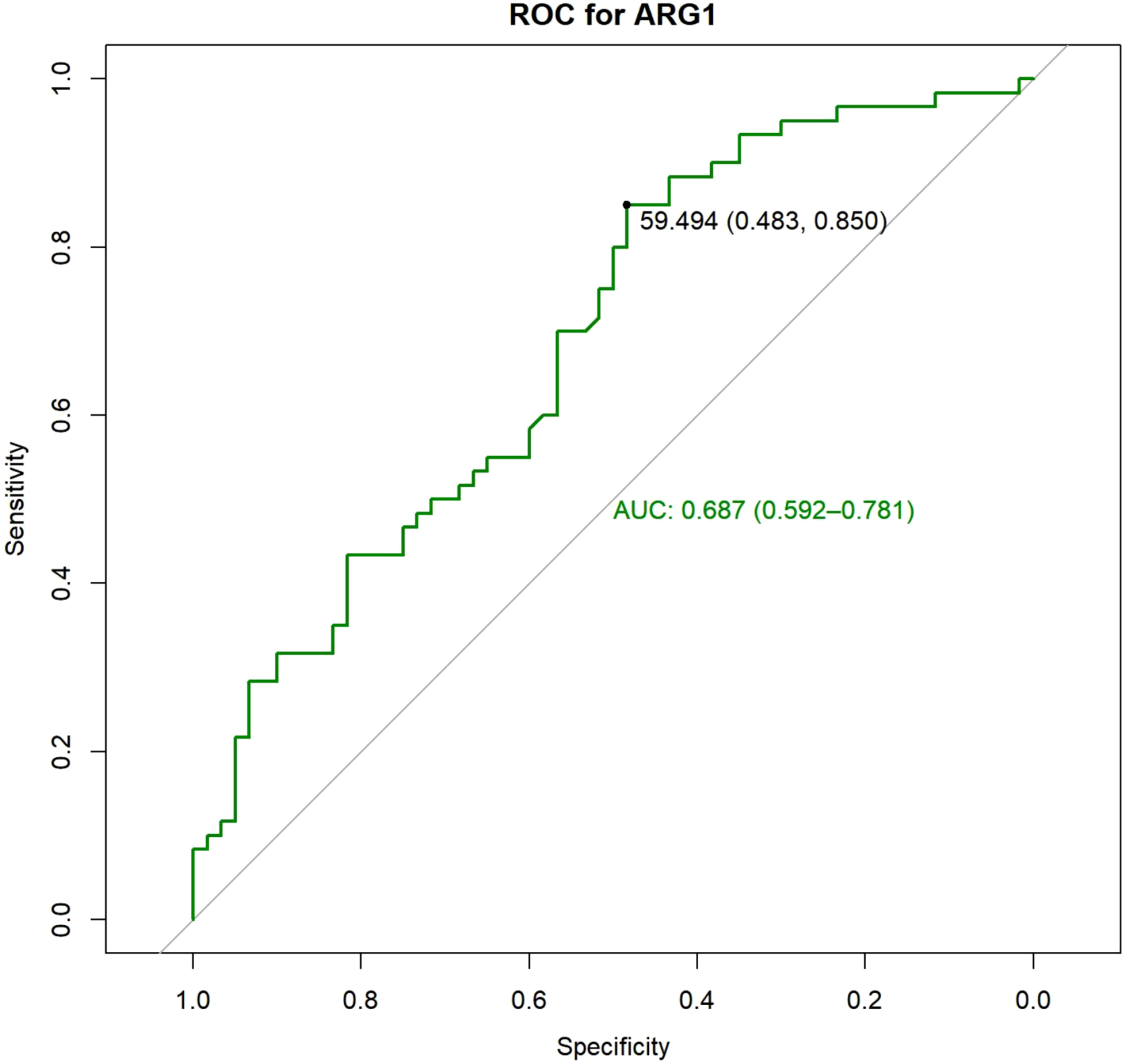
ROC analysis was performed and the best cut-off value was determined to be 59.49 ng/ml, at which both sensitivity and specificity were high.

A total of 228 patients were enrolled in the study. Till December 2020, 200 (200/228, 87.71%) patients developed TACE refractoriness during follow-up and were included in the analysis, whereas 28 were excluded. The patients were divided into a training set and a validation set according at a ratio of 3:2. The ARG1 levels in the training and validation sets were 45.38 ± 28.07 ng/mL and 45.38 ± 27.68 ng/mL, respectively. There was no significant difference in the expression of ARG1 between the two groups (P=1.000). In the training set, the area under the ROC curve of ARG1 was 0.687 (0.592-0.781), the best cut-off value was 59.49 ng/mL, the sensitivity was 48.3%, and the specificity was 85.0% (**Figure 1**). The ARG1 expression levels were further used to classify the patients as belonging to the high expression group (>59.49 ng/mL) and the low expression group (<59.49 ng/mL).

### Baseline characteristics of the patients

There was no statistically significant difference in the baseline data of the training and validation sets of the patients (**Table 1**). There were 120 patients in the training set, of which 60 patients showed TACE refractoriness within 12 months of follow-up (early TACE refractoriness), indicating an incidence rate of 50%. The incidence of early TACE refractoriness in the validation set was 48.75%, and there was no statistically significant difference between the two groups (P=0.888). All baseline characteristics of patients in training set and validation set were not significantly different (P>0.05).

**Table 1:** Baseline characteristics of patients with TACE refractoriness in training set and validation set.

Variables	Training set	Validation set	*P*-value
	N = 120	N = 80	
Sex n (%)			0.944
Male	114 (95.0)	77 (96.2)	
Female	6 (5.0)	3 (3.8)	
Age(years)	55.48 ± 11.15	54.05 ± 10.28	0.362
Hepatitis n(%)			0.368
None	2 (1.7)	4 (5.0)	
HBV	90 (75.0)	56 (70.0)	
HCV	28 (23.3)	20 (25.0)	
BCLC n(%)			0.751
A	59 (49.2)	42 (52.5)	
B	61 (50.8)	38 (47.5)	
Child-Pugh n(%)			0.539
5	49 (40.8)	39 (48.8)	
6	60 (50.0)	35 (43.8)	
7	11 (9.2)	6 (7.5)	
Tumor number n(%)			0.885
single	56 (46.7)	39 (48.8)	
multiple	64 (53.3)	41 (51.2)	
Tumor size, cm	7.12 ± 3.66	7.51 ± 3.27	0.448
Tumor position			0.544
Single	58 (48.3)	43 (53.8)	
Double	62 (51.7)	37 (46.2)	
ARG1, n(%)			0.736
<59.49 ng/mL	82 (68.3)	52 (65.0)	
>59.49 ng/mL	38 (31.7)	28 (35.0)	
AFP, n(%)			0.748
<400 ng/mL	68 (56.7)	48 (60.0)	
≥ 400 ng/mL	52 (43.3)	32 (40.0)	
Ascites n(%)			0.974
None	90 (75.0)	59 (73.8)	
Have	30 (25.0)	21 (26.2)	
RBC, ×10^12^/L	4.66 (1.18)	4.45 (1.47)	0.258
PLT,×10^9^/L	144.16 (45.69)	140.24 (39.86)	0.533
ALT, U/L	43.96 (24.20)	47.76 (26.93)	0.299
AST, U/L	33.43 (15.27)	33.04 (14.06)	0.853
TBIL, mmol/L	21.71 (11.68)	21.03 (10.88)	0.681
ALB, g/L	38.66 (5.14)	39.62 (5.41)	0.206

BCLC, Barcelona clinic liver cancer; AFP, alpha-fetoprotein; RBC, red blood cell; PLT, platelet; ALT, alanine aminotransferase; AST, aspartate aminotransferase; TBIL, total bilirubin; ALB, albumin.

In the training set, 95% of the patients were male with an average age of 55.48 ± 11.15 years. Viral hepatitis was the main cause of HCC (98.3%), including infection with hepatitis B or C virus. Patients with single tumors accounted for approximately 46.7% of all patients and those with multiple tumors accounted for 53.3%; among the patients with multiple tumors, those with 2–3 tumors accounted for approximately 28.1%, and those with ≥ 4 tumors accounted for approximately 25.2%. The average size of the largest tumor in all patients was 7.12 ± 3.66 cm. According to the BCLC staging criteria, the proportion of patients with stages A and B was 49.2% and 50.8%, respectively. TACE refractoriness was mainly characterized by new intrahepatic lesions (71.7%), vascular invasion (20.0%) and extrahepatic lesions (8.3%).

### Prognostic factors affecting patients with early TACE refractoriness

Univariate analysis of the predictors of early TACE refractoriness showed that BCLC stage (OR=2.110, 95%CI=1.014-4.350, P=0.046), tumor size (OR=1.202, 95% CI=1.074-1.346, P=0.001), occurrence of multiple tumors (OR=3.471, 95% CI=1.635-7.370, P=0.001), platelet count (OR =0.987, 95%CI=0.979-0.995,P=0.003), alanine aminotransferase levels (OR=1.017, 95%CI= 1.001-1.033,P=0.035), and high ARG1 levels (OR=0.189, 95% CI=0.079-0.451, P<0.001) were predictors of early TACE refractoriness. Among them, high platelet count and high ARG1 levels were protective factors against early TACE refractoriness. Multivariate analysis showed that tumor size (OR =1.138, 95%CI=1.006–1.288, P=0.041), occurrence of multiple tumors (OR=4.374, 95% CI = 1.189–16.089, P=0.026), platelet count (OR =0.99, 95%CI=0.980–0.999, P=0.036), and high ARG1 levels (OR=0.209, 95%CI=0.079–0.551, P=0.002) were independent predictors of early TACE refractoriness (**Table 2**).

**Table 2:** Univariate and multivariate analyses for predictive factors of early TACE refractoriness.

Variables	Univariate analysis			Multivariate analysis		
	OR	95% CI	*P* value	OR	95% CI	*P* value
Gender	1.000	0.194-5.165	1.000			
Age	1.018	0.985-1.052	0.284			
Hepatitis-B	0.957	0.058-15.768	0.975			
Hepatitis-C	1.154	0.065-20.342	0.922			
BCLC	2.110	1.014-4.350	0.046	0.378	0.103–1.381	0.141
Child-Pugh score = 6	1.312	0.615-2.797	0.482			
Child-Pugh score = 7	2.148	0.556-8.296	0.268			
Multiple tumors	3.471	1.635-7.370	0.001	4.374	1.189–16.089	0.026
Tumor size, cm	1.202	1.074-1.346	0.001	1.138	1.006–1.288	0.041
Tumor position	1.307	0.637-2.678	0.465			
ARG1>59.49 ng/mL	0.189	0.079-0.451	<0.001	0.209	0.079–0.551	0.002
AFP > 400 ng/mL	0.762	0.369-1.571	0.462			
Ascites	1.429	0.622-3.285	0.400			
RBC, ×10^12^/L	1.206	0.885-1.644	0.235			
PLT,×10^9^/L	0.987	0.979-0.995	0.003	0.990	0.980–0.999	0.036
ALT, U/L	1.017	1.001-1.033	0.035	1.015	0.996-1.034	0.115
AST, U/L	0.994	0.971-1.018	0.614			
TBIL, mmol/L	1.002	0.972-1.034	0.888			
ALB, g/L	1.031	0.961-1.106	0.395			

BCLC, Barcelona clinic liver cancer; AFP, alpha-fetoprotein; RBC, red blood cell; PLT, platelet; ALT, alanine aminotransferase; AST, aspartate aminotransferase; TBIL, total bilirubin; ALB, albumin.

### Establishment of a predictive model for early TACE refractoriness based on plasma ARG1 levels

**Figure 2:**
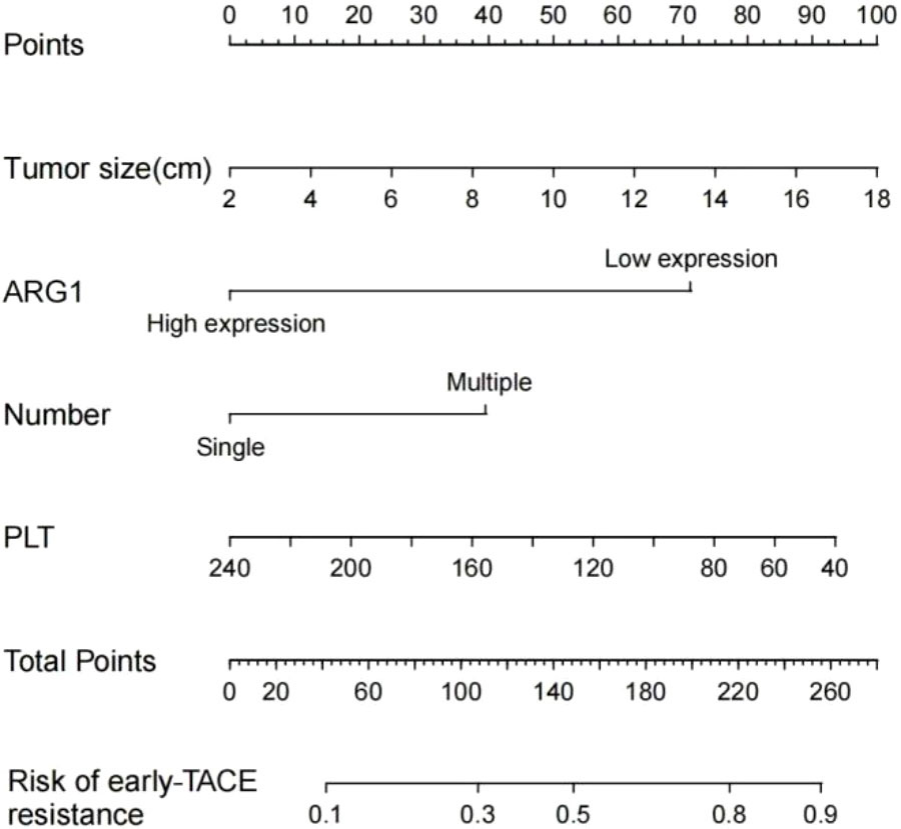
Prognostic nomogram for early TACE refractoriness. Each value of the patient is indicated on the axis with the variable. A line is drawn upward to determine the number of points received by each variable. Then, the total number of points are calculated and a line is drawn downward to determine the prediction probability of early TACE refractoriness.

We established a nomogram based on the significant predictors identified by binary logistic regression analysis of the univariate and multivariate analysis data (**Figure 2**), including those on tumor size, tumor number, platelet count, and ARG1 expression. The AUC value obtained for the nomogram of the training cohort was 0.786 (95% CI=0.702–0.870), and the validation set AUC value was 0.833 (95% CI=0.791–0.875), indicating a good diagnostic value and some significance for prediction of early TACE refractoriness in individuals.

**Figure 3:**
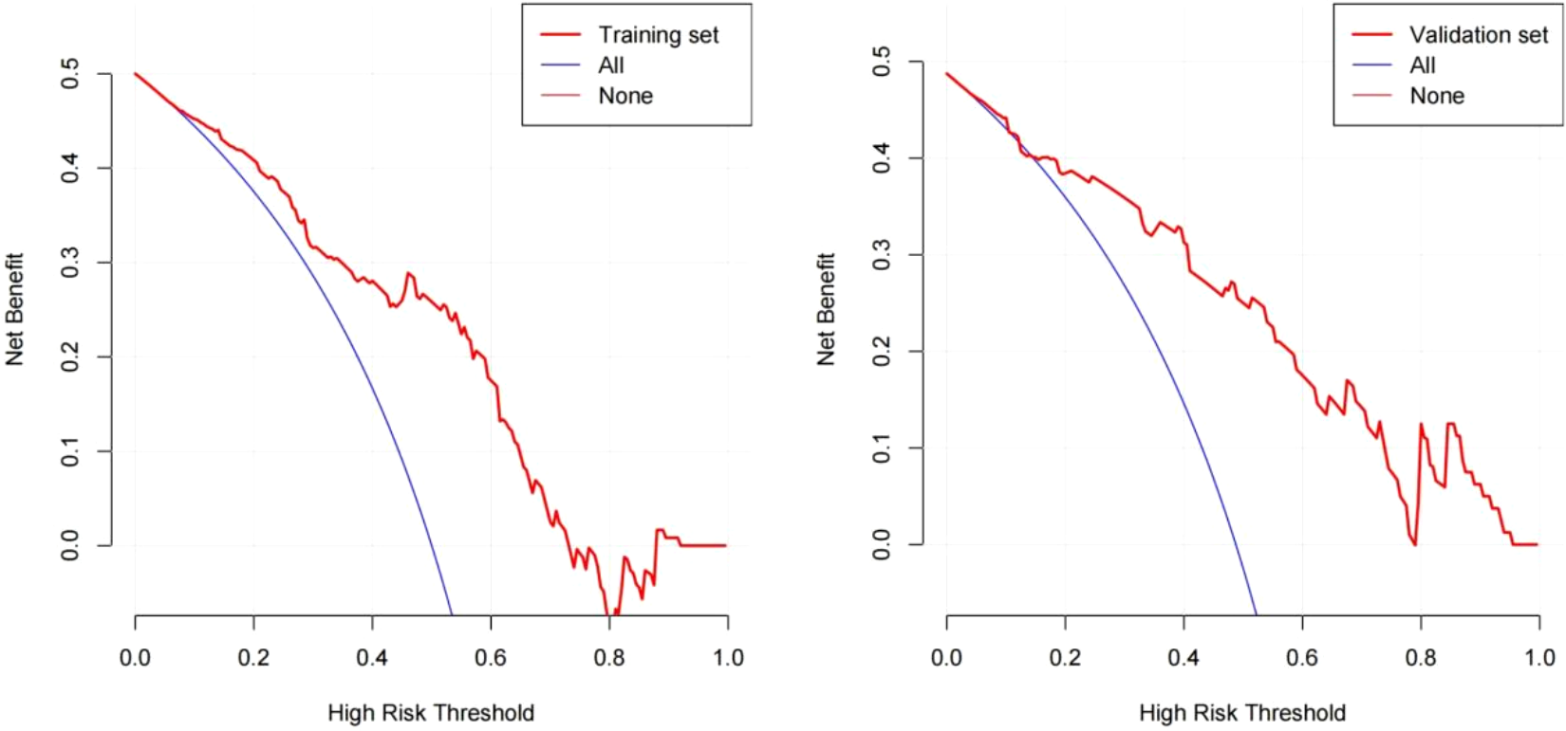
Decision curve analysis for the nomogram based on clinical characteristics. The red polyline represents the nomogram. The horizontal line with an ordinate of 0 represents all negative samples. For these participants, the treat-none scheme was applied. The blue fine line represents all positive samples, and for these participants, the treat-all scheme was applied.

The DCA result for the nomogram is presented in **Figure 3**. In the training set, the decision curve demonstrated that if the threshold probability of a patient or physician was between 9% and 75%, using the developed nomogram to predict the incidence of early TACE refractoriness was more beneficial, than when using the treat-all-scheme or treat-none schemes. Therefore, the developed nomogram was more beneficial than the treat-all scheme or the treat-none scheme for decision-making regarding treatment administration. Further, the validation set showed better results.

### Presentation of a patient with early TACE refractoriness

**Figure 4:**
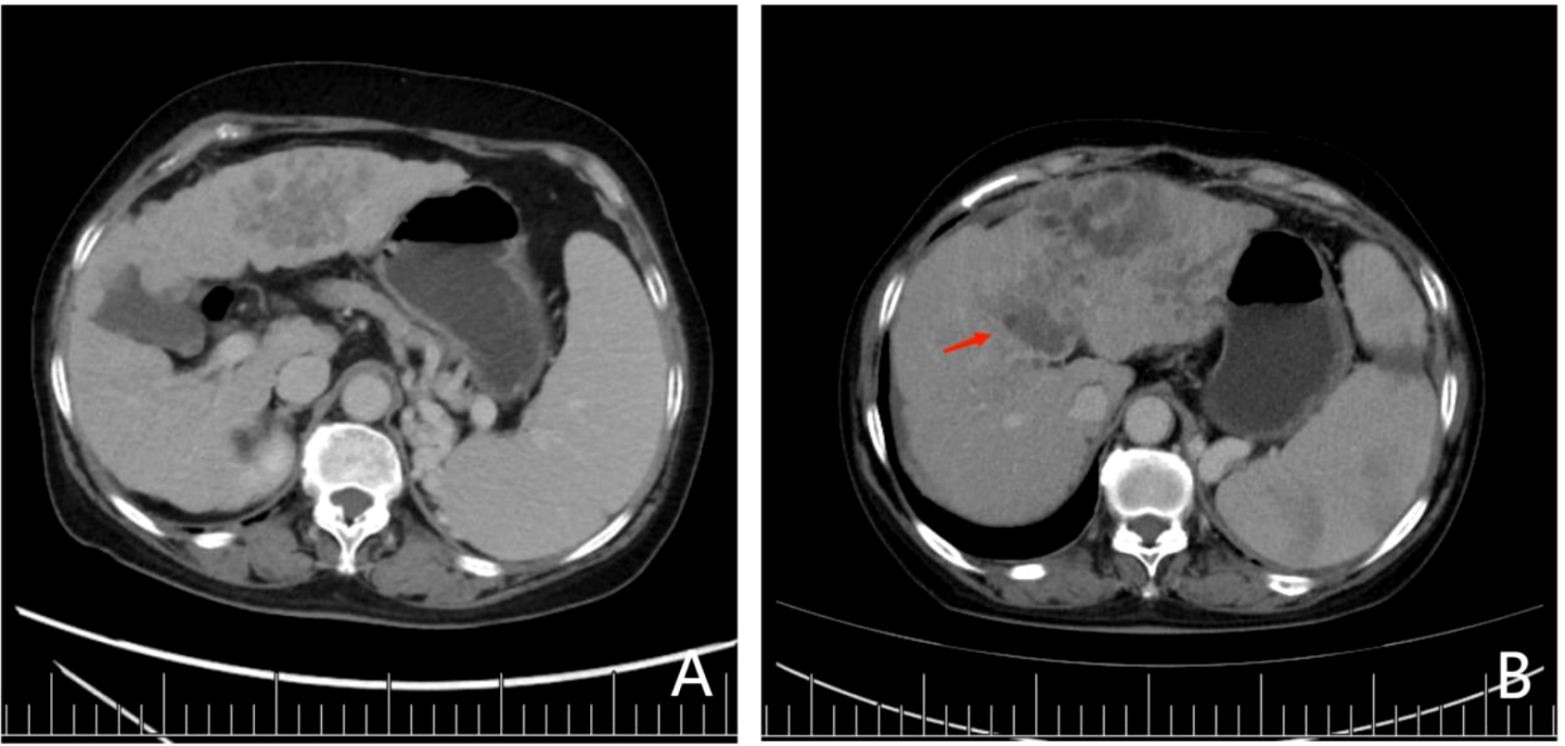
**(A)** Preoperative contrast-enhanced CT showed two lesions in the patient’s liver with cirrhosis. The largest lesion was 7.42 cm in diameter, located in the left lobe of the liver. In contrast-enhanced CT, lesions in the arterial phase were significantly enhanced, although they were weakened in the delayed phase, which was in accordance with the diagnostic criteria for hepatocellular carcinoma. Portal vein blood flow was unobstructed, and no portal vein tumor thrombus was found. **(B)** After 2 cycles of TACE treatment, lesions in the left hepatic lobe were enlarged, and tumor thrombus was seen in the portal vein (red arrow).

The clinical data of a 56-year-old female patient with hepatocellular carcinoma has been presented here. The platelet count, tumor size, number of lesions, and plasma ARG1 levels of the patient were 67.7×109/L, approximately 7.42 cm, 2, and 22.6 ng/mL, respectively. The above data were consistent with the nomogram-total score of 220, which corresponds to a probability of early TACE resistance of approximately 82% (**Figure 2**). The CT image results from March 2020 are shown in **Figure 4A**; two TACE treatments were performed in March and May 2020. In August 2020, the patient developed a portal vein tumor thrombus (**Figure 4B**), which indicated disease progression. Furthermore, disease progression occurred 5 months after the patient’s first TACE treatment, suggesting that the patient developed early TACE refractoriness. Predicted results of the nomogram were consistent with the observed condition of the patient.

## Discussion

To our knowledge, this was the first study to report the correlation between plasma ARG1 levels and the efficacy of TACE in unresectable HCC patients. This study found that plasma ARG1 expression was an independent predictor of early TACE refractoriness. We developed a nomogram based on ARG1 expression levels, combined with different tumor parameters, to predict the risk of early TACE refractoriness in unresectable HCC patients treated with TACE. Incorporation of ARG1 expression and the associated clinical prognostic factors into an easy-to-use nomogram could facilitate an individualized assessment of the risk of developing early TACE refractoriness preoperatively.

Recent studies have confirmed that ARG1 can be induced in alternately activated (M2) macrophages and is involved in the occurrence and development of tumors, mainly due to the anti-inflammatory response, tumor immunity, tumor proliferation, metastasis, and immunosuppression (15). Therefore, altered expression of ARG1 may lead to changes in the metabolism of the liver tissue and have marked effects on the metabolic and growth statuses of tumor cells. Previous studies have shown that expression of ARG1 was closely related to HCC differentiation, histological type, Edmondson grade, and other indicators that indicate the degree of tumor differentiation. With a decrease in ARG1 expression, the differentiation degree of HCC worsened, suggesting that ARG1 may be a molecular marker for determining the degree of HCC differentiation (10, 16).

Our study found that the average ARG1 level of 60 patients with early TACE refractoriness was 36.55 ng/mL and that of patients without early TACE was 54.22 ng/mL; the former value was significantly lower than the latter, and the difference was statistically significant (P < 0.05). In unresectable HCC patients treated with TACE, the risk of early TACE refractoriness decreased with an increase in ARG1 expression, possibly because the increased ARG1 expression increased the catalysis and consumption of arginine and reduced the amount of arginine in the microenvironment (17). Hepatoma cells are malignant tumor cells with arginine deficiency. Arginine is an essential amino acid. The growth of cancer cells is inhibited in the absence of arginine. A reduction in the levels of amino acids in HCC tissues inhibits the growth and reproduction of tumor cells, improves the efficacy of TACE, and reduces the risk of tumor progression and TACE refractoriness after repeated TACE. If exogenous arginine is administered to the tumor microenvironment to deplete arginine, tumor growth can be inhibited. Currently, the antitumor drug PEGylated recombinant human arginase is being tested in clinical trial including patients with unresectable HCC (18).

However, the effect of ARG1 on patient survival remains controversial. Obiorah et al. reported that patients with high ARG1 expression have a shorter median time to recurrence (19), whereas Mao et al. found that patients with low ARG1 expression in HCC had shorter DFS and OS (11). Our study showed that the expression of plasma ARG1 in unresectable HCC patients is negatively correlated with the probability of early TACE refractoriness, that is, the higher the expression of plasma ARG1, the lower the risk of early TACE refractoriness and the better the prognosis. Our results were similar to Mao’s findings, suggesting that the assessment of plasma ARG1 levels would be helpful in assessing the risk of early TACE refractoriness in patients with HCC treated with TACE.

There is no consensus yet regarding the risk factors affecting TACE refractoriness, and the results of various studies differ widely. By studying the molecular indicators and clinical biochemical indicators, some researchers found that microRNA, TP53 mutation, the M2 isoform of pyruvate kinase expression level, AFP level, and interleukin-8 expression level can affect the outcome of TACE (14, 20–22). Shehta et al. found thrombocytopenia to possibly be an important predictor of tumor recurrence after hepatectomy in HCC patients with cirrhosis, which is similar to our findings (23). However, the pathophysiological mechanism remains to be explored further. The results of our study showed that BCLC stage, tumor size, the presence of multiple tumors, platelet count, and plasma ARG1 level as shown by our univariate analyses may be predictive factors for early TACE refractoriness. However, in multivariate analyses we found that only tumor number, tumor size, and platelet and plasma ARG1 levels were independent predictive factors for early TACE refractoriness. Moreover, our univariate analyses indicated that BCLC and ALT were predictors of early TACE refractoriness. However, our multivariate analyses of these factors instead yielded statistically insignificant associations. One possible explanation for this seeming contradiction is that since tumor size and number were both strongly correlated with BCLC stage, they therefore may also be related to ALT. In other words, we managed to obtain a complete result from analyzing only a single factor. And by eliminating the influences of other factors *via* our multivariate analyses, we revealed that neither BCLC stage nor ALT independently influence the prediction of early TACE refractoriness.

In recent years, many studies have attempted to establish a predictive model for the prognosis and response to TACE treatment. However, relatively few studies have used TACE refractoriness as an endpoint (24, 25). Current research mainly focuses on predicting postoperative response to TACE, and there are very few studies on the early prediction of TACE refractoriness, most of them being retrospective, focusing on the relationship between a certain index and TACE refractoriness. Furthermore, only a few predictive models integrate multiple clinical indicators. Existing models are mostly based on local samples and lack clinical and external validations (26). In this study, we developed a nomogram based on ARG1 expression level, combined with different tumor parameters, to predict the risk of early TACE refractoriness in patients with unresectable HCC. The logistic regression model included ARG1, tumor size and number, and platelet counts, and the results showed the model c-index to be higher (0.833), indicating that models incorporating ARG1 expression level had higher prediction accuracy. The DCA suggested that the nomogram had good clinical utility. Many previous studies have thoroughly discussed the relationships between tumor size, number, and hepatic physiology-related indicators and TACE efficacy. Increases in either tumor size or number lead to increased |tumor burdens that markedly influence liver cancer treatment outcomes (20, 27–29). Hu et al. retrospectively analyzed the association between TACE refractoriness and various biomarkers; they proposed that the main risk factors for refractory TACE include AFP, some liver function indicators and tumor imaging findings (30). Taken together, these previous results are consistent with our findings.

Our study had some limitations. First, we used the definition of TACE refractoriness provided by the JSH, which is applicable in clinical practice; however, since TACE treatment itself is highly heterogeneous, the concept of “refractoriness” has certain limitations and presently, there is no international consensus regarding its definition. Second, this is a single-center retrospective study with a small sample size, which may lead to potential data selection bias. Therefore, the results of this study may need to be further verified by prospective, multicenter, large sample, randomized controlled trials. Third, this predictive model is temporarily not applicable to patients with advanced HCC or those receiving TACE combined with other treatments. The correlation between ARG1 and TACE refractoriness in these specific patient populations will be the topic of our continued research in this direction.

In conclusion, to our knowledge, we showed for the first time that the expression of plasma ARG1 in unresectable HCC patients is negatively correlated with the probability of early TACE refractoriness. The expression of plasma ARG1 before TACE in patients with unresectable HCC can be used as one of the candidate biomarkers for predicting early TACE refractoriness. A nomogram based on tumor size, tumor number, platelet, and plasma ARG1 levels could help predict the possibility of early TACE refractoriness before TACE treatment to achieve an individualized prediction of early TACE refractoriness in different patients.

## Data availability statement

The raw data supporting the conclusions of this article will be made available by the authors, without undue reservation.

## Ethics statement

The ethics of the research program has been approved by the Ethics Committee the Affiliated Cancer Hospital of Zhengzhou University and Henan Cancer Hospital review board. The ethics committee waived the requirement of written informed consent for participation.

## Author contributions

Conceptualization and design the study: H-LL and H-TH; Provision of study materials or patients: W-LX, LZ, D-YZ, Q-JY, and C-YG; Collection and assembly of data: W-LX, YG, X-HZ, and S-JX; Data analysis and interpretation: X-HZ, YG, YZ,LZ, and D-YZ; Manuscript writing: W-LX; Manuscript reviewing: H-LL, H-TH, and W-JF; Final approval of manuscript: All authors.

## Funding

This work was supported by The National Natural Science Foundation (82002596), Henan Province Natural Science Foundation (212300410403),Science and Technology Department of Henan Province (No. 212102310162); Medical Science and Technology Research Project of Henan Province (No. LHGJ20190633) and Technology Major Project of the Ministry of Science and Technology of China (2018ZX10303502).

## Acknowledgments

Thanks to all patients and medical staff who participated in the study.

## Conflict of interest

The authors declare that the research was conducted in the absence of any commercial or financial relationships that could be construed as a potential conflict of interest.

## Publisher’s note

All claims expressed in this article are solely those of the authors and do not necessarily represent those of their affiliated organizations, or those of the publisher, the editors and the reviewers. Any product that may be evaluated in this article, or claim that may be made by its manufacturer, is not guaranteed or endorsed by the publisher.
